# Dedifferentiated soft tissue leiomyosarcoma with heterologous osteosarcoma component: case report and review of the literature

**DOI:** 10.1186/s13569-020-00129-5

**Published:** 2020-04-05

**Authors:** Raffaele Gaeta, Davide Matera, Francesco Muratori, Giuliana Roselli, Giacomo Baldi, Domenico Andrea Campanacci, Alessandro Franchi

**Affiliations:** 1 Department of Translational Research and of New Technologies in Medicine and Surgery, University of Pisa, Via Paradisa 2, 56124 Pisa, Italy; 2 Department of Orthopaedic Oncology and Reconstructive Surgery, Azienda Ospedaliera Universitaria Careggi, Florence, Italy; 3 Department of Diagnostic Imaging, Azienda Ospedaliera Universitaria Careggi, Florence, Italy; 4 Department of Oncology, “S. Stefano” Hospital, Prato, Italy

**Keywords:** Soft tissues, Leiomyosarcoma, Dedifferentiated, Osteosarcoma

## Abstract

**Background:**

Soft tissue dedifferentiated leiomyosarcoma with heterologous osteosarcomatous component is an extremely rare entity described in only few cases in the literature.

**Case presentation:**

We report the case of a 65-year-old male patient who, after initial inadequate surgery of a tumor of the left forearm, developed local recurrence that was treated with neoadjuvant chemotherapy, surgery and postoperative radiation therapy. Histologically the tumor showed an abrupt separation of two different patterns. One component consisted of interlacing fascicles of spindle cells with cigar-shaped nuclei strongly positive for smooth muscle actin, desmin and H-caldesmon. The other component consisted of a high-grade pleomorphic sarcoma with osteoid and chondroid matrix production, which positive for SATB2. Thus, a final diagnosis of dedifferentiated leiomyosarcoma was rendered. Fifteen months after treatment, the patient presented further local and distant relapse with pulmonary metastases and died 23 months after the first presentation.

**Discussion and conclusions:**

Dedifferentiated leiomyosarcoma is a highly malignant neoplasm with a poor outcome. Extensive sampling of soft tissue leiomyosarcomas is recommended to detect possible dedifferentiated areas as they represent a crucial prognostic parameter.

## Background

Leiomyosarcoma (LMS) is an aggressive soft tissue sarcoma that accounts for about 7 to 10% of all soft tissue sarcomas and usually occurs in adult and elderly patients [1]. The most common involved site is the retroperitoneum [2], but it can also affect internal organs (e.g. uterus or stomach), whereas localization at the limbs is unusual [3]. Moreover, sinonasal tract LMSs are exceptionally rare with no more than 100 cases reported in the English literature [4]. LMS can also occur in bone, as primary or secondary tumor localization from distant sites, although the former is rare, with about 0.7% incidence of all primary malignant bone tumors [5].

The term ‘dedifferentiated leiomyosarcoma’ was first used by Schmookler and Lauer in 1983 [6] and refers to a tumor morphologically characterized by an abrupt transition from classic leiomyosarcoma to high-grade sarcoma which does not express immunohistochemical muscular markers. In rare cases, the non-myogenic component of the tumor presents heterologous differentiation. Here we describe a case of a leiomyosarcoma with an osteosarcomatous dedifferentiated component and we review the clinico-pathologic features of the previously reported cases.

## Case presentation

**Fig. 1:**
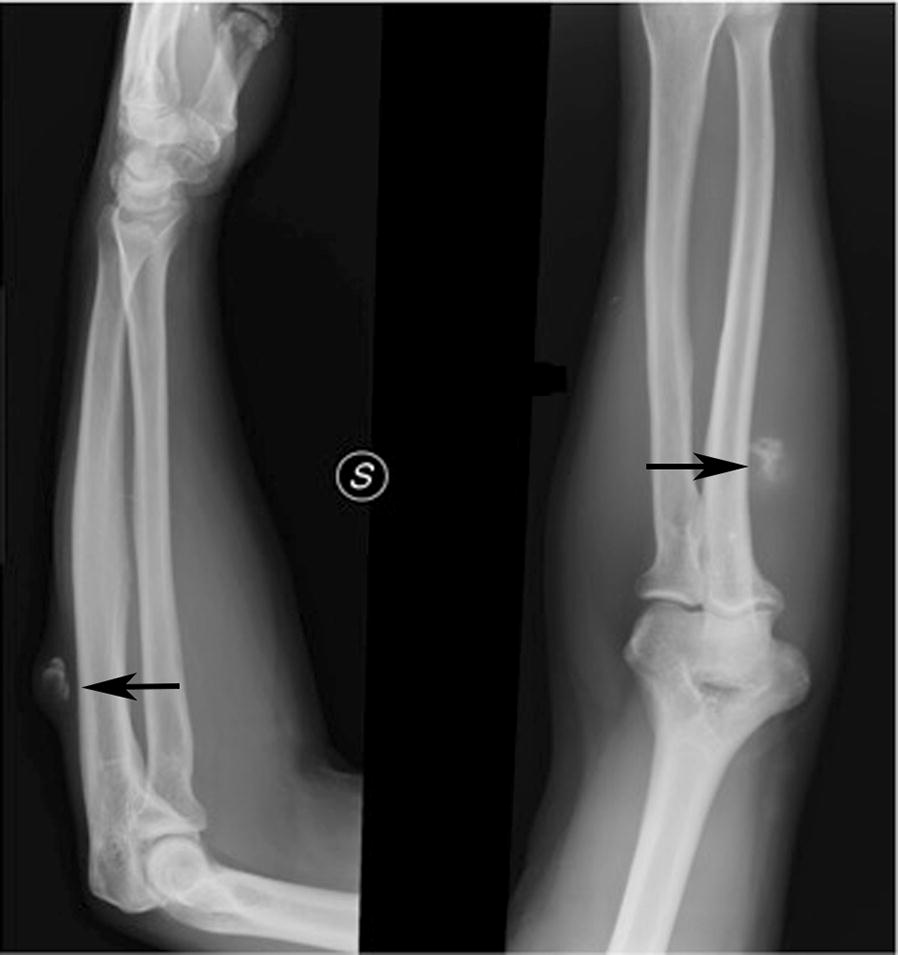
X-ray of the forearm showing a soft tissue tumor with areas of calcification (arrows)

**Fig. 2:**
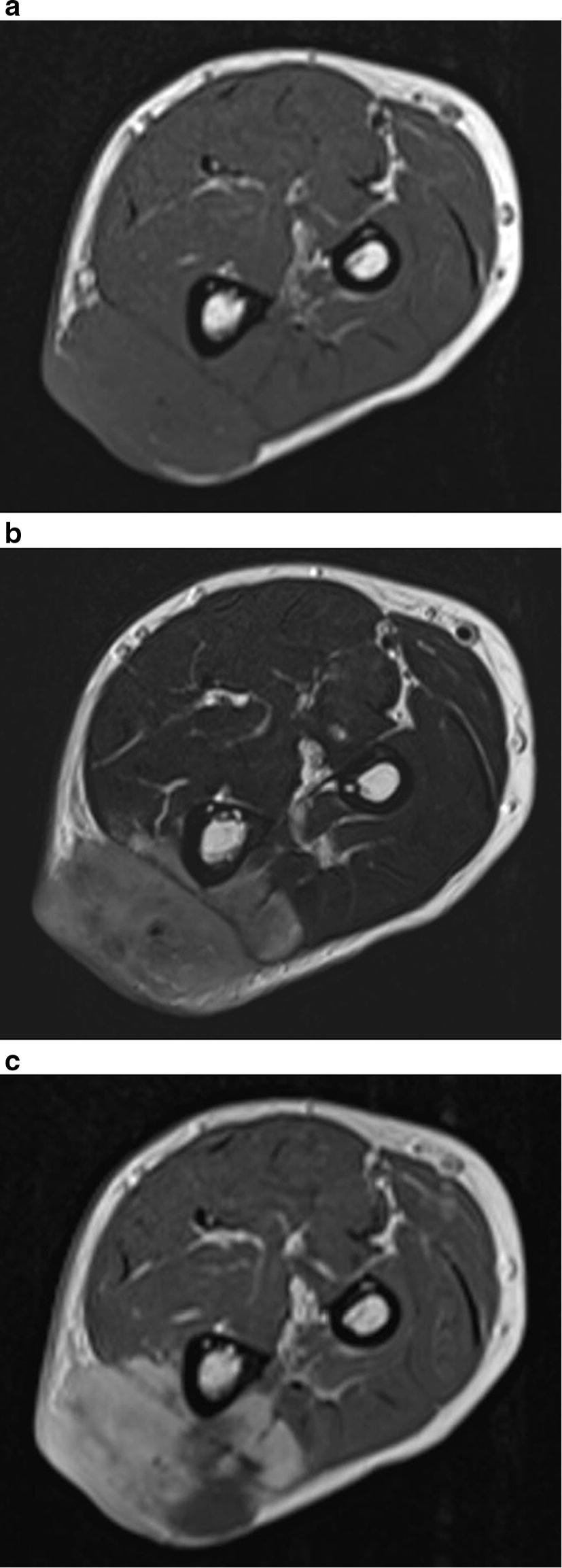
Magnetic Resonance Imaging (MRI) showing a soft tissue mass in the subcutis. The lesion is isointense to muscle in T1-weighted sequence (**a**), hyperintense on T2-weighted sequence (**b**), and shows contrast medium enhancement on T1-weighted sequence (**c**)

**Fig. 3:**
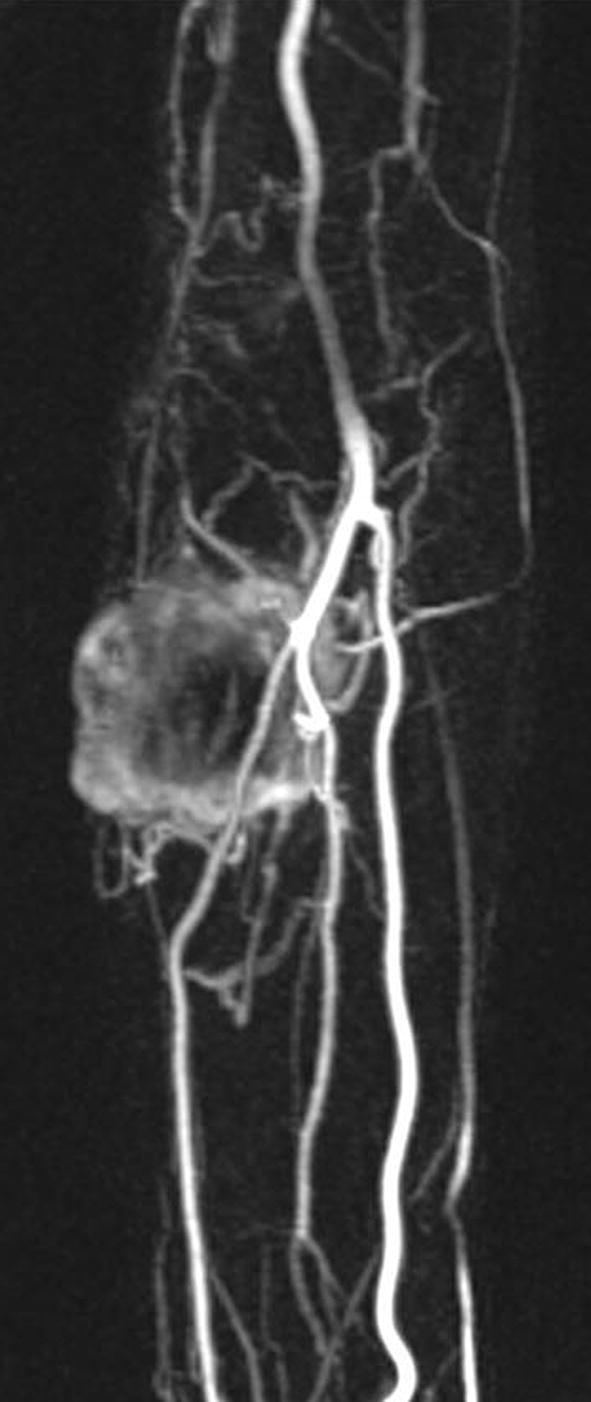
Maximum intensity projections with dynamic contrast enhanced magnetic resonance imaging (DCE-MRI) showing high vascularization in the arterial phase

A 65-year-old man came to our observation with a solid-elastic mass of about 4.5 cm maximum diameter on the proximal region of the left forearm, ulnar side, that appeared hypomobile on the underline planes. He reported being affected by this “bump” for at least 20 years, but it had recently shown a rapid growth. Furthermore, he reported having already undergone another excision in local anesthesia after an ultrasound examination about 1 year before at another hospital. An X-ray of the forearm showed a soft tissue mass with irregular calcifications (Fig. 1) and CT scan of the limb confirmed this finding. Magnetic Resonance Imaging (MRI) showed a soft tissue mass arising in the subcutis and involving the skin and the subfascial and muscular planes, with partial invasion of wrist and fingers flexor muscles and partially surrounding the bone (lateral side of the ulna). The mass was isointense to muscle in T1-weighted sequence (Fig. 2a), hyperintense on T2-weighted sequence (Fig. 2b), with contrast medium enhancement on T1-weighted sequence (Fig. 2c) and highly vascular (Fig. 3). Staging exams (total body Computed Tomography scan and Positron Emission Tomography scan), showed no other tumor localizations.

After preoperative needle biopsy, surgical excision of the tumor was performed, including skin (with the scar of the previous surgery), fascia, muscles and a portion of the ulna. Reconstruction was performed with plate, screws and cement and coverage with a microsurgical rotation flap.

**Fig. 4:**
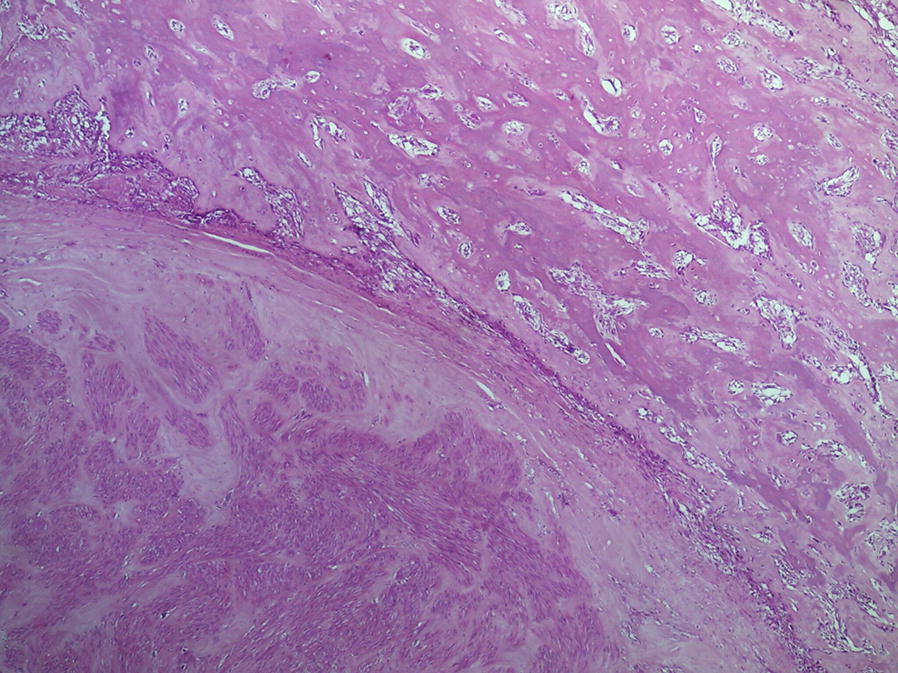
Low power view showing the two abruptly separated components of the tumor. Well-differentiated leiomyosarcoma is shown in the lower and high-grade osteosarcoma in the upper right (40×)

**Fig. 5:**
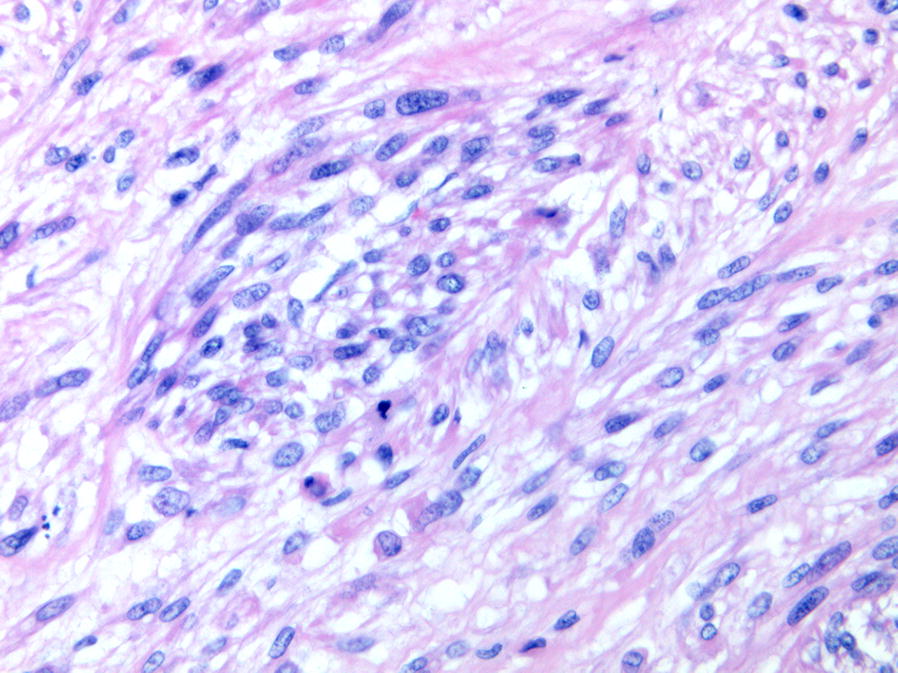
Well-differentiated leiomyosarcoma consisting of spindle cells arranged in intersecting fascicles (200×)

**Fig. 6:**
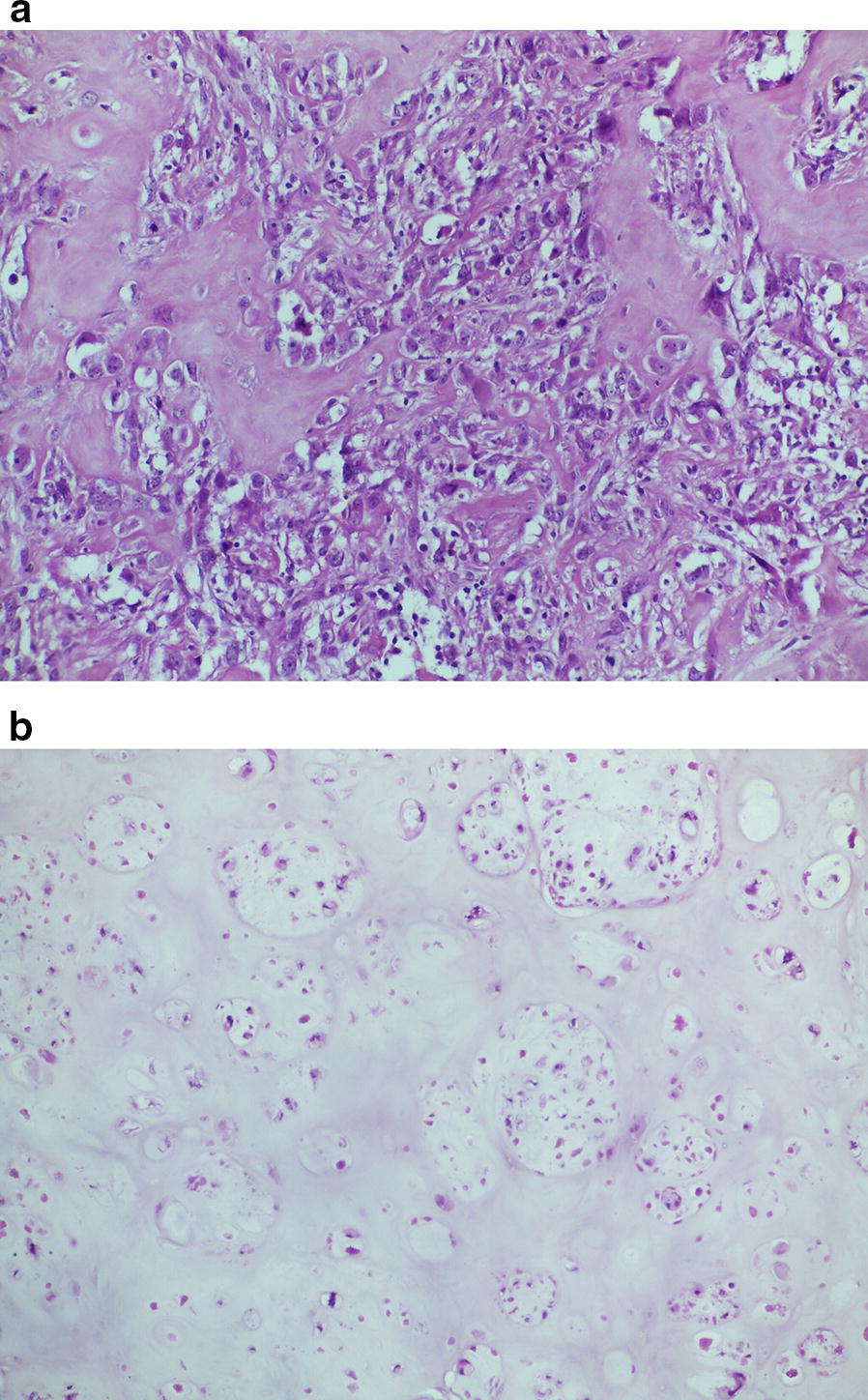
High-grade osteosarcoma consisting of highly atypical cells with osteoid matrix production (**a**; 200×). In other areas neoplastic cells produced cartilage matrix (**b**; 100×)

Gross examination of the surgical specimen revealed a solid white neoplasm with a maximum diameter of 6 cm in the soft tissues. Histologically, the tumor showed two different, abruptly separated components (Fig. 4). One consisted of a proliferation of atypical spindle or ovoid cells with cigar-shaped nuclei and eosinophilic cytoplasm arranged in long interlacing fascicles, an appearance consistent with leiomyosarcoma (Fig. 5). The other component consisted of atypical pleomorphic cells producing osteoid matrix in disorganized trabeculae with focal calcification (Fig. 6). Areas of cartilage matrix production were identified as well.

**Fig. 7:**
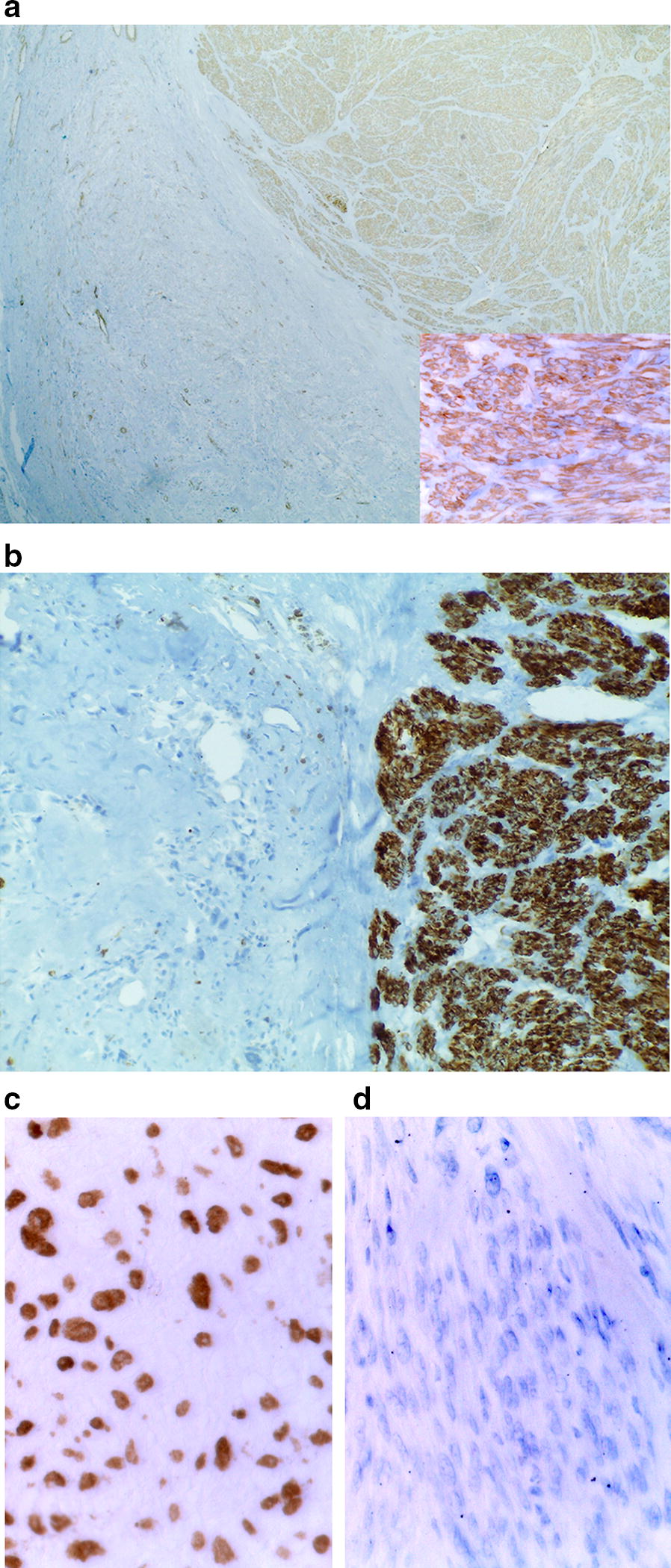
The leiomyosarcoma component (on the right in **a** and **b**) is positive for alpha smooth muscle actin (**a**, 40×; inset 400×) and desmin (**b**, 100×), whereas the osteosarcoma component (on the left in **a** and **b**) is negative. SATB2 was positive in the nuclei of neoplastic cells in the osteosarcoma component (**c**, 400×) and negative in the leiomyosarcoma (**d**, 400×)

Immunohistochemically, areas with leiomyosarcomatous appearance were strongly positive for alpha smooth muscle actin (Fig. 7a) and desmin (Fig. 7b), whereas the osteosarcomatous component was negative. Both components were negative for cytokeratins (AE1/AE3 and CAM5.2), EMA, S100 protein, CD34 and CD31. SATB2, a marker of osteoblastic differentiation was positive only in the osteosarcomatous component, whereas leiomyosarcoma areas were negative (Fig. 7c, d).

The final diagnosis was leiomyosarcoma grade 2, according to the French Federation of Comprehensive Cancer Centers (FNCLCC) grading system [7] with areas of high-grade osteosarcoma (so-called dedifferentiated leiomyosarcoma). The tumor infiltrated the dermis, the subcutis and the striated muscle. Surgical margins were free of tumour. The review of the histologic slides from the previous surgical excision showed similar histological aspects, although the tumor consisted mainly of leiomyosarcoma with a very limited osteosarcomatous component.

After the surgical treatment, the patient received three cycles of high-dose adjuvant chemotherapy with adriamycin and iphosphamide and local sequential radiotherapy (66 Gy). After an interval of 15 months, MRI showed multiple local recurrences at the site of previous excisions and a PET CT total body scan highlighted bilateral pulmonary nodules, for which he underwent a new cycle of chemotherapy with gentamicin. The patient died after 23 months from presentation due to the progression of the disease.

## Discussion and conclusions

Even if there is no consensus regarding the exact definition of dedifferentiated leiomyosarcoma, it may be described as a well-differentiated tumor showing typical features of a smooth muscle neoplasm with a high-grade undifferentiated pleomorphic sarcoma component lacking expression of muscle markers.

Dedifferentiation is a well-known occurrence in liposarcoma and chondrosarcoma, but this phenomenon has been only rarely reported in LMS. In most cases, dedifferentiated LMS presents discrete transition from well-differentiated smooth muscle morphology to high-grade pleomorphic morphology with loss of smooth muscle differentiation [8]. Only exceptionally the dedifferentiated component shows heterologous osteosarcomatous differentiation, and review of the literature revealed only four previously reported extrauterine tumors (Table 1). The search of the literature also included the term ‘malignant mesenchymoma’ that has been abandoned because it is ambiguous.

**Table 1:** Summary of the salient clinico-pathologic features of dedifferentiated leiomyosarcomas with osteosarcomatous elements of the peripheral soft tissue reported in the literature

Case n.	Age	Sex	Primary site	Local recurrence	Metastases	Status	References
1	71	M	Leg	Yes	Lung	Deceased after 22 months	[9]
2	35	F	Groin	Yes	Lung	Unknown	[10]
3	66	M	Retroperitoneum	Yes	Liver	Deceased after 8 months	[8]
4	60	M	Thigh	Yes	Occipital and lung	Unknown	[11]
5	65	M	Forearm	Yes	Lung	Deceased after 23 months	Present case

The first case was a 71-year-old man who underwent resection of an intramuscular lesion of the leg, which had rapidly enlarged in the preceding 8 weeks [9]. The tumour was excised widely and postoperative local radiotherapy was administered. A local recurrence and a solitary pulmonary metastasis were excised 8 and 9 months later respectively. The patient died of disseminated disease 22 months after initial excision [9].

Grabellus et al. [10] studied a soft tissue tumor of the right groin region of a 35-year-old woman and an initial diagnosis of high grade LMS was made. Lung metastases were discovered 6 months after the first surgical procedure and 7 months later, the tumor showed local recurrence. Histologically, the recurrence showed high cellularity and was composed by round irregular to pleomorphic cells with high mitotic activity. Neoplastic osteoid was deposited in a lacy, trabecular pattern and was focally mineralized. The tumor cells resembled malignant osteoblasts.

The large series of 18 dedifferentiated LMSs reported by Chen et al. [8] included a retroperitoneal mass composed by well-differentiated LMS with numerous osteoclastic giant cells and heterologous osteosarcomatous differentiation with malignant osteoid formation. The patient was a 66-year old man treated with neoadjuvant radiation prior to surgical excision followed by chemotherapy, but he developed hepatic metastases and died of disease after 8 months. One further retroperitoneal LMS in this series presented benign-appearing bone and cartilage. This patient was alive with disease 37 months after presentation.

Feeley et al. [11] described a 60-year old man with a LMS in the left thigh. The lesion was resected but he presented 2 years later with pulmonary relapse and, 18 months later, with metastases in the occipital lobe. This lesion showed extensive osteoid deposition surrounded by pleomorphic, mitotically active spindle cells, consistent with osteosarcomatous differentiation. This is considered an unusual pattern of tumour progression, in which a well-differentiated LMS developed divergent osteosarcomatous differentiation in the recurrence.

The main clinical and histological differential diagnosis is with myositis ossificans. This benign lesion presents a characteristic zonal growth pattern, with a central area that consists of a nodular fasciitis-like proliferation of bland spindle cells, and progressive deposition of osteoid matrix in the periphery. Malignant bone forming tumors of the soft tissues, including the present case, lack this orderly growth pattern and usually present marked cytologic atypia. Another entity that can be considered in the differential diagnosis is dedifferentiated liposarcoma with heterologous smooth muscle and osteo-chondroblastic differentiation. This possibility was excluded because there was no evidence of a well-differentiated liposarcoma component in both the first specimen and in the recurrence, and in addition the tumor was negative for MDM2 and CDK4. The possibility that the lesion represents a soft tissue osteosarcoma in which spindle cell areas show features of leiomyosarcoma or myofibrosarcoma was also evaluated, but it seemed unlikely because the two components were always sharply separated, a feature more consistent with the concept of dedifferentiation.

Considering all the cases reported, the average age is 60 years, with a clear male predominance (4:1). It is very important to notice that all the 5 patients had local recurrences and developed distant metastases despite aggressive treatments. In 3 patients the outcome was fatal (death at 8, 22 and 23 months after surgical excision) due to the progression of the disease, while no clinical follow-up was detailed for the remaining 2 patients. These data confirm that dedifferentiated LMS of the soft parts is a highly aggressive tumor. According to the literature, these neoplasms present 50% to 65.2% of mortality and 89% of incidence of metastasis [12, 13]. It has also been reported that the loss of myogenic markers in LMS may be a significant prognostic factor, accounting for a significantly higher aggressiveness [14]. For these reasons, extensive sampling is recommended in soft tissue LMS in order to detail the different components that may be present.

## Data Availability

The datasets during and/or analysed during the current study available from the corresponding author on reasonable request.
